# Challenges and recommendations of vaccination in immunosuppression

**DOI:** 10.61622/rbgo/2025FPS7

**Published:** 2025-09-16

**Authors:** Caroline Alves de Oliveira Martins, Isabella Ballalai, Juarez Cunha, Susana Aidé

**Affiliations:** 1 Universidade Federal Fluminense Rio de Janeiro RJ Brazil 1-Universidade Federal Fluminense, Rio de Janeiro, RJ, Brazil; Comissão Nacional Especializada de Vacinas da Federação Brasileira das Associações de Ginecologia e Obstetrícia (Febrasgo), Brazil.; 2 Federação Brasileira das Associações de Ginecologia e Obstetrícia Brazil 2-Comissão Nacional Especializada de Vacinas da Federação Brasileira das Associações de Ginecologia e Obstetrícia (Febrasgo); Sociedade Brasileira de Imunizações (SBIm), Brazil.; 3 Federação Brasileira das Associações de Ginecologia e Obstetrícia Brazil 3-Comissão Nacional Especializada de Vacinas da Federação Brasileira das Associações de Ginecologia e Obstetrícia (Febrasgo); Brasileira de Imunizações (SBIm), Brazil.; 4 Universidade Federal Fluminense Rio de Janeiro RJ Brazil 4-Universidade Federal Fluminense, Rio de Janeiro, RJ, Brazil; Comissão Nacional Especializada de Vacinas da Federação Brasileira das Associações de Ginecologia e Obstetrícia (Febrasgo), Brazil.

## Abstract

•Address the types of immunodeficiency and the greater susceptibility to severe infections compared to the general population, as well as a less efficient response to vaccine stimuli.

•Provide information on the negative impacts of infections on the health of immunodeficient individuals and their complications.

•Provide knowledge of studies on the efficacy and safety of vaccines in the immunosuppressed population.

•Clarify which vaccines should be indicated, the best time to administer them, and when to revaccinate.

•Update gynecologists and obstetricians on the vaccination schedule for this population and on the position of the Brazilian Federation of Gynecology and Obstetrics Associations (Febrasgo) regarding the vaccines made available by the National Immunization Program, including by the Reference Centers for Special Immunobiologicals (Portuguese acronym: CRIEs) and private vaccination services.

**Recommendations**

- Febrasgo understands the negative impact of infections on the health of immunocompromised individuals and their complications.
- Febrasgo monitors the published evidence on the various vaccines and has the role of bringing evidence-based knowledge from studies on the efficacy and safety of vaccines in the immunocompromised population.
- Febrasgo believes the best way to contain the large volume of misinformation and fake news about vaccines is to provide gynecologists and obstetricians with constant updates on the best scientific evidence available.
- Febrasgo’s National Specialized Commission on Vaccines understands that in order to reduce deaths, hospitalizations and negative impacts on families and health systems, it is necessary to take advantage of every opportunity to inform and update health professionals on the benefits and risks of vaccines and vaccine-preventable diseases, so they can make the necessary choices and recommendations for their immunosuppressed patients.
- Febrasgo contends that providing robust and precise information can significantly address vaccine hesitancy and, consequently, reduce morbidity and mortality from vaccine-preventable pathogens, especially in the immunosuppressed population.

## Background

Innovations in the field of pharmaceuticals have expanded therapeutic options and increased life expectancy for patients with diseases associated with varying degrees of immunodeficiency. These advances include treatments such as radiotherapy, chemotherapy, immunotherapy, targeted therapy, immunobiologicals, and other medications used in oncological, rheumatological, dermatological, and anti-inflammatory conditions.^(1,2)^ Consequently, there has been a significant increase in the demand for immunizations beyond the basic vaccination schedule. This article discusses the strategies adopted in Brazil for the vaccination of immunocompromised individuals due to disease or treatment, highlighting the resources made available by the Unified Health System (SUS) through the Reference Centers for Special Immunobiologicals (Portuguese acronym: CRIEs)^(3)^, and the vaccination schedules developed by national institutions. The Brazilian Society of Immunizations (Portuguese acronym: SBIm) constantly prepares and updates specific schedules for the vaccination of special patients.^(4)^ Febrasgo produced in its series of guidelines and recommendations, through the National Specialized Commission on Vaccines, the Vaccination Program for Women with a chapter dedicated to the vaccination of immunocompromised women.^(5)^ Like all protocols, the manuals mentioned above are constantly updated, considering new knowledge of immunobiologicals incorporated into both basic schedules and those for special patients.^(6)^

### What is the definition of immunodeficiency?

In this article, immunocompromised people are those distinguished from the general population by their inability to respond to numerous antigenic or infectious stimuli, which makes them more susceptible to infections of varied natures, generally with greater severity than in the general population, and to respond less efficiently to vaccine stimuli.^(3,7,8)^

### What are the variables found in the immunization of immunocompromised people?

Some variables in the immunization of immunocompromised individuals are the host response to the immunogen, different degrees of immunocompromise and whether it is permanent or temporary, in addition to the risk of applying attenuated viral vaccines to this group. Studies on immunogenicity and efficacy for vaccines in this group are scarce both for qualitative response (production of functional antibodies) and quantitative response (level of antibodies produced).^(3,6,8)^ This scientific gap highlights the need for more robust and specific research to develop safe and effective vaccination protocols for these populations.

### What is the evidence on the benefits of vaccines in immunocompromised individuals? What data regarding safety is available in the literature?

The use of inactivated vaccines in immunocompromised individuals generally does not pose an additional risk of adverse effects. However, in general, there are few publications and studies quantifying their benefits. On the other hand, live attenuated virus vaccines, the vaccine virus, even if very “weakened”, can cause adverse events related to increased viral replication. Its indication will depend on the epidemiological situation, when the risk of the natural disease and its complications clearly exceed the risks of vaccine complications for that type of immunosuppression.^(6,8,9)^ Therefore, referral of the patient to the CRIE or private service with a report presenting the diagnosis, medications in use or current and/or planned treatments is essential to adequately guide the health professional at the CRIE, the Basic Health Unit (UBS) or the private service responsible for vaccination.^(6,10)^

### What is the best time to prescribe vaccines?

Vaccination with inactivated vaccines should preferably be updated up to two weeks before starting immunosuppressive therapy and, when using attenuated vaccines, four weeks earlier. If it is not possible to use the inactivated vaccine before starting immunosuppressive therapy, there is no contraindication to its use during treatment. However, the benefits are affected and, in some cases, the vaccine must be repeated after the period of immunosuppression. The period after stopping the immunosuppressive drug varies, depending on the clinical condition and the type of treatment applied.^8,11)^

The immunogenicity and efficacy of the hepatitis B vaccine in immunocompromised patients are lower compared to healthy individuals. For this reason, four doses of hepatitis B vaccine with twice the usual dose are recommended, as well as the evaluation of the vaccine response.^(3,4)^ More recently licensed vaccines, such as herpes zoster, which include a potent adjuvant in their formulation, were able to generate immunological responses and efficacy similar to that found in the non-immunocompromised group for most situations.^(10-14)^

### How are immunodeficiencies classified?

They are classified as primary or secondary. Primary immunodeficiencies are related to congenital immunodeficiencies of both innate and acquired immunity, comprising a wide variety of clinical conditions. Secondary immunodeficiency is caused by the underlying disease itself (infections, malignancies, autoimmune diseases, among others) and its treatment with drugs that cause varying degrees of immunocompromise.^(6)^ Secondary immunodeficiency is the most common.^(2,3)^

### Vaccination for inborn errors of immunity

Diseases related to inborn errors of immunity vary according to the specific alterations. They can be divided into: deficiency of humoral immunity (B cells), deficiency of cellular immunity (T cells), combined deficiency of humoral immunity (B cells) and cellular immunity (T cells), complement deficiency and deficiency of phagocytic function.

The type and severity of immunodeficiency guide the indication of immunobiologicals: attenuated vaccines should not be indicated in severe immunodeficiencies, while inactivated vaccines should be indicated even if they do not induce immunological responses similar to those of immunocompetent individuals, as they can still benefit individuals.^(6)^

In general, live attenuated vaccines should not be administered to individuals with cellular immunodeficiency. Live attenuated and inactivated vaccines may be recommended for patients with isolated immunoglobulin deficiencies. Patients with complement deficiency should receive vaccines that protect against encapsulated germs, since infections with such germs (Neisseria meningitidis, Streptococcus pneumoniae, and Haemophilus influenzae) are a high risk for these patients. Patients with phagocytosis deficiency should not receive live bacterial vaccines, such as BCG (Bacillus Calmette and Guérin), but can receive all other vaccines.^(3)^

Specially recommended vaccines: Whenever possible, pneumococcal conjugate vaccine 20 or 15 (PCV20 or PCV15) should be used. If not possible, PCV13 with or without sequential use of the polysaccharide vaccine (PPV23). When PCV20 is used, there is no indication for a sequential regimen with PPV23. Haemophilus influenzae type b, meningococcal ACWY conjugate, meningococcal B, hepatitis A and B, influenza (preferably the high-dose [HD] vaccine in people aged 60 and over), COVID-19, herpes zoster from 18 years of age, human papillomavirus (HPV), respiratory syncytial virus (RSV) from 60 years of age and over.^(3,4)^ There are two RSV vaccines available: Arexvy® (GsK) and Abrysvo® (Pfizer). Abrysvo® is licensed by Anvisa for people with certain chronic medical conditions, from 18 to 59 years of age. It is also the only one licensed for pregnant women.

Unavailable in CRIEs: PCV15 and PCV20, meningococcal B, herpes zoster, HD influenza, HPV9 and RSV.^(3,4)^

Other recommended vaccines: Adult-type double bacterial (Td) or, preferably, adult-type acellular triple bacterial (Tdap).^(3,4)^

Contraindications: Attenuated vaccines – BCG, yellow fever, triple viral (measles, mumps and rubella), tetraviral (measles, mumps, rubella and varicella), varicella and dengue – may be recommended according to the individual assessment of the patient, with restrictions:

- In the presence of combined deficiencies of cellular and humoral immunity, all are contraindicated;
- In the presence of severe humoral immunity deficiencies or phagocytosis deficiencies (chronic granulomatous disease), BCG is contraindicated;
- In the presence of IgA deficiency and immunoglobulin subclasses or complement deficiencies, attenuated vaccines are not contraindicated.^(3,4)^

### Vaccination when using immunosuppressive drugs

#### Corticosteroids

High levels of immunosuppression are considered to be present with daily doses of corticosteroids > 20 mg for 14 days or more, which contraindicates the use of attenuated vaccines during this period. The interval between the suspension of corticosteroids with immunosuppressive doses and the administration of attenuated vaccines is one month (Table 1). During treatment with corticosteroids in a therapeutic regimen that is not considered immunosuppressive, such as in physiological doses, low-potency topical use in localized areas of the skin, inhaled, in the conjunctiva and intra-articular injections, the use of attenuated vaccines is not contraindicated.^(6)^

**Table 1:** Drugs that can cause immunocompromise and the interval between the discontinuation of treatment and the application of attenuated vaccines

Drugs	Immunosuppressive Dose	Vaccination interval
Corticosteroids (Prednisone or equivalent)	≥ 2 mg/kg/day or ≥ 20 mg/day for more than two weeks	One month
Methotrexate	≥ 0.4 mg/kg/week; ≥ 20 mg/day	One to three months
Leflunomide	0.25-0.5 mg/kg/day; ≥ 20 mg/day	When serum levels are below 0.02 mg/L
Sulfasalazine and hydroxychloroquine	_	None
Mycophenolate mofetil	3 g/day	Three months
Azathioprine	1-3 mg/kg/day	Three months
Cyclophosphamide	0.5-2.0 mg/kg/day	Three months
Cyclosporine	>2.5 mg/kg/day	Three months
Tacrolimus	0.1-0.2 mg/kg/day	Three months
6-mercaptopurine	1.5 mg/kg/day	Three months
Biologicals: anticytokines and T-lymphocyte costimulation inhibitors	_	Three months, a minimum of five half-lives, or whichever is shorter
B-lymphocyte depleting biologicals	_	Six months
Target-specific synthetics: JAK inhibitors (Tofacitinib)	_	Two weeks

Notes: 1. Vaccinate preferably before immunosuppression. Inactivated vaccines should be administered at least 14 days before starting immunosuppressive therapy and live attenuated vaccines, ideally four weeks prior. If it is not possible to wait, maintain a minimum interval of two weeks. 2. Babies of women who used biologicals during pregnancy: live attenuated vaccines can be administered after 6-8 months of age. Source: Adapted from Brazilian Society of Immunizations (2025).^(4)^

#### Biological immunosuppressants

Biologicals are products based on monoclonal antibodies, cell fusion proteins, anti-interleukins and T lymphocyte co-stimulation blockers, which inactivate or block specific targets such as cells, cytokines or other immune mediators. They are indicated for the treatment of immune-mediated conditions, such as rheumatoid arthritis, inflammatory bowel disease, among others. Their action can last weeks to months after discontinuation. The degree of immunosuppression varies according to the drug, dose and duration of treatment. They are generally used in conjunction with other immunosuppressants such as methotrexate and corticosteroids.^(15-17)^

Specially recommended vaccines: Whenever possible, PCV20 or PCV15 should be used and if not possible, PCV13 with or without sequential use of the pneumococcal polysaccharide vaccine (PPV23). When PCV20 is used, there is no indication for a sequential regimen with PPV23. Haemophilus influenzae type b, meningococcal conjugate C or ACWY (whenever possible), meningococcal B, hepatitis A and B, influenza (preferably the high-dose vaccine [HD] in people aged 60 and over), COVID-19, herpes zoster from 18 years of age, HPV and RSV from 60 years.^(3,4)^

There are two RSV vaccines available: Arexvy® (GsK) and Abrysvo® (Pfizer). Abrysvo® is licensed by Anvisa for people with certain chronic medical conditions from 18 to 59 years of age. It is also the only one licensed for pregnant women.

Unavailable in CRIEs: PCV15 and PCV20; meningococcal ACWY (available in some conditions) and meningococcal B; herpes zoster; HD influenza, HPV9 and RSV.^(3,4)^

Other recommended vaccines: Adult-type double bacterial (Td) or, preferably, adult-type acellular triple bacterial (Tdap).^(3,4)^

Contraindications: Live attenuated virus vaccines – BCG, yellow fever, triple viral (measles, mumps and rubella), tetraviral (measles, mumps, rubella and varicella), varicella and dengue.^(3,4)^

For safety, live attenuated virus vaccines should be administered 14 to 30 days before introduction and only three to six months after the end of immunosuppressive therapy. They can be administered three months after chemotherapy, but at least six months after therapy with anti-B cell antibodies (rituximab) (Table 1).

Recommendations for vaccination of infants born to mothers who used immunosuppressants during pregnancy should be considered by the attending physician. To determine the appropriate immunosuppressive dose, the dose administered per kilogram of the pregnant woman’s weight during the entire period of use should be considered, in addition to the period of pregnancy in which it was used.^(4)^

Non-biological immunosuppressants

Several medications, depending on the dose used, can act as immunosuppressants, such as methotrexate, cyclosporine, tacrolimus, mycophenolate mofetil, azathioprine, leflunomide and 6-mercaptopurine. The CRIE manual recommends a three-month interval after discontinuation of these drugs for the use of attenuated vaccines.^(6,16)^Table 1 shows the drugs that can cause immunocompromise and the interval between the discontinuation of treatment and the application of attenuated vaccines, taken from the SBIm Special Patients Schedule.^(4)^

Specially recommended vaccines: Whenever possible, PCV20 or PCV15 should be used and if not possible, PCV13 with or without sequential use of the pneumococcal polysaccharide vaccine (PPV23). When PCV20 is used, there is no indication for a sequential regimen with PPV23. Haemophilus influenzae type b, meningococcal conjugate C or ACWY (whenever possible), meningococcal B, hepatitis A and B, influenza (preferably the high-dose vaccine [HD] in people aged 60 and over), COVID-19, herpes zoster from 18 years of age, HPV and RSV from 60 years.^(3,4)^

There are two RSV vaccines available: Arexvy® (GsK) and Abrysvo® (Pfizer). Abrysvo® is licensed by Anvisa for people with certain chronic medical conditions from 18 to 59 years of age. It is also the only one licensed for pregnant women.

Unavailable in CRIEs: PCV15 and PCV20; meningococcal ACWY and meningococcal B; herpes zoster; HD influenza, HPV9 and RSV.^(3,4)^

Other recommended vaccines: Adult-type double bacterial (Td) or, preferably, adult-type acellular triple bacterial (Tdap).^(3,4)^

Contraindications: Live attenuated virus vaccines – BCG, yellow fever, triple viral (measles, mumps and rubella), tetraviral (measles, mumps, rubella and varicella), varicella and dengue.

#### Vaccination in people living with HIV/AIDS

Currently existing studies on the safety and efficacy of vaccines in people living with HIV/AIDS do not yet allow for the establishment of conduct free from controversy. In the face of HIV infection, the great heterogeneity in situations is also clear, from immunocompetence at the beginning of the infection, to severe immunodeficiency with the progression of the disease.^(3,18)^

People living with HIV/AIDS can receive all the vaccines recommended in the schedules as early as possible, before they present clinical signs or severe immunodeficiency.^(6)^ As immunosuppression increases, the risk of administering live attenuated vaccines also increases, as does the possibility of an insufficient or inadequate immune response.^(18)^

In the case of severe immunodeficiency, the administration of vaccines should be postponed until a satisfactory degree of immune reconstruction is achieved with the use of antiretroviral therapy, aiming at improving the vaccine response and reducing the risk of post-vaccination complications. The administration of live attenuated vaccines to immunocompromised patients should be subject to individual risk-benefit analysis and should not be performed in cases of severe immunocompromise.^(3,18)^Chart 1 presents the levels of immunocompromise.^(4)^

**Chart 1:** Levels of immunocompromise of people living with HIV/AIDS according to the CD4+ T lymphocyte count (CD4+)

Levels of immunocompromise from 13 years of age	
Immune alteration	CD4+ T lymphocytes count in cells per mm^3^
Small or absent (1)	≥ 350
Moderate (2)	Between 200 and 500
Severe (3)	< 200

Source: Adapted from the Brazilian Society of Immunizations (2025).^(4)^

Specially recommended vaccines: Whenever possible, PCV20 or PCV15 should be used and if not possible, PCV13 with or without sequential use of the pneumococcal polysaccharide vaccine (PPV23). When PCV20 is used, there is no indication for a sequential regimen with PPV23. Haemophilus influenzae type b, meningococcal conjugate ACWY, meningococcal B, hepatitis A and B, influenza (preferably the high-dose vaccine [HD] in people aged 60 and over), COVID-19, herpes zoster from 18 years of age, HPV and RSV from 60 years.^(3,4)^

There are two RSV vaccines available: Arexvy® (GsK) and Abrysvo® (Pfizer). Abrysvo® is licensed by Anvisa for people with certain chronic medical conditions from 18 to 59 years of age. It is also the only one licensed for pregnant women.

Unavailable in CRIEs: PCV15 and PCV20; meningococcal B; herpes zoster; HD influenza, HPV9 and RSV.^(3,4)^

Other recommended vaccines: Adult-type double bacterial (Td) or, preferably, adult-type acellular triple bacterial (Tdap).^(3,4)^

Vaccines depending on CD4 levels: Live attenuated virus vaccines: triple viral, varicella, yellow fever, dengue. The decision to indicate yellow fever will depend on CD4 levels and the risk in the region.^(3,4)^

#### Vaccination in people with active oncological disease

As in other immunosuppressive situations, vaccination should be indicated before starting treatment. Live attenuated virus vaccines should be administered 14 to 30 days before introduction and only three to six months after the end of immunosuppressive therapy. The use of attenuated vaccines is not recommended during the period of treatment with chemotherapy and/or radiotherapy. Inactivated vaccines can be administered during treatment, but they must be repeated after the treatment is completed, as there is no way to ensure a good immune response.^(3,6,19-21)^

The deadline for receiving attenuated and inactivated vaccines is three months after the end of immunosuppressive therapy, and six months for those who used anti-B cell antibodies, such as rituximab.^(6,21)^

Specially recommended vaccines: Whenever possible, PCV20 or PCV15 should be used and if not possible, PCV13 with or without sequential use of the pneumococcal polysaccharide vaccine (PPV23). When PCV20 is used, there is no indication for a sequential regimen with PPV23. Haemophilus influenzae type b, meningococcal conjugate C or ACWY (whenever possible), meningococcal B, hepatitis A and B, influenza (preferably the high-dose vaccine [HD] in people aged 60 and over), COVID-19, herpes zoster from 18 years of age, HPV and RSV from 60 years.^(3,4)^

There are two RSV vaccines available: Arexvy® (GsK) and Abrysvo® (Pfizer). Abrysvo® is licensed by Anvisa for people with certain chronic medical conditions from 18 to 59 years of age. It is also the only one licensed for pregnant women.

Unavailable in CRIEs: PCV15 and PCV20; meningococcal ACWY and meningococcal B; herpes zoster; HD influenza, HPV9 and RSV.^(3,4)^

Other recommended vaccines: Adult-type double bacterial (Td) or, preferably, adult-type acellular triple bacterial (Tdap).^(3,4)^

Contraindications: Live attenuated virus vaccines – BCG, yellow fever, triple viral (measles, mumps and rubella), tetraviral (measles, mumps, rubella and varicella), varicella and dengue.^(3,4)^

#### Vaccines in solid organ transplant recipients

Candidates for solid organ transplants should have their vaccination schedules evaluated and updated. The justification is the immunosuppressive activity of the underlying disease (e.g., chronic renal failure, neoplasms) and immunosuppressive therapy after the transplant.^(3,17)^

Vaccination of donors should be considered so that they do not constitute a source of transmission of vaccine-preventable diseases to the recipient. Their vaccination should be guided sufficiently in advance so that the vaccination schedules can be carried out and an effective immune response can occur before the transplant, justifying, in some circumstances, the shortening of the vaccination schedule, which should be completed, if possible, up to 14 days before the transplant.^(3,17,20)^

Specially recommended vaccines: Whenever possible, PCV20 or PCV15 should be used and if not possible, PCV13 with or without sequential use of the pneumococcal polysaccharide vaccine (PPV23). When PCV20 is used, there is no indication for a sequential regimen with PPV23. Haemophilus influenzae type b, meningococcal conjugate ACWY, meningococcal B, hepatitis A and B, influenza (preferably the high-dose vaccine [HD] in people aged 60 and over), COVID-19, herpes zoster from 18 years of age, HPV, dual bacterial adult-type (Td) or triple bacterial acellular adult-type (Tdap) and RSV from 60 years.^(3,4)^

There are two RSV vaccines available: Arexvy® (GsK) and Abrysvo® (Pfizer). Abrysvo® is licensed by Anvisa for people with certain chronic medical conditions from 18 to 59 years of age. It is also the only one licensed for pregnant women.

Unavailable in CRIEs: PCV15 and PCV20; meningococcal B; herpes zoster; HD influenza, HPV9 and RSV.^(3,4)^

Vaccines especially recommended before transplantation (in the case of immunocompetent patients): Yellow fever, triple viral (measles, mumps and rubella), tetraviral (measles, mumps, rubella and varicella) and dengue.^(3,4)^ These live attenuated virus vaccines should be administered up to four weeks before the transplant.

#### Vaccines in people who have undergone hematopoietic stem cell transplants

All vaccines administered before hematopoietic stem cell transplantation should be repeated.

Several factors alter the immune response in transplant recipients: the donor’s immunity, the type and time after the transplant, and the associated immunosuppressive treatment.

There are two types of transplants: allogeneic (external donor) and autologous (the donor is the individual himself). In the first case, there is immunocompromise due to factors such as chemotherapy, T-cell suppression protocols and graft-versus-host disease, which further depress the patient. In the second case, there is chemotherapy with or without radiotherapy, although there is no immunosuppressive therapy after the cell infusion.

Neutropenia occurs in the first 30 days after the transplant, with the possibility of bacterial and fungal infections, so immunization is not recommended. Between 30 and 100 days after the transplant, infections by cytomegalovirus, varicella-zoster, pneumococcus and Haemophilus influenzae type b occur, increasing the risk in the presence of graft-versus-host disease. After 100 days, considered the late post-transplant period, the risks are similar to those of the previous period. Immunity is gradually restored and immunocompetence is considered to be reestablished approximately two years after transplantation, in the absence of graft-versus-host disease and immunosuppressive therapy.^(22)^

For allogeneic transplants donors, vaccination with inactivated vaccines should be completed at least 14 days before transplantation, and live vaccines at least 30 days prior. This timeframe allows sufficient time for immunity transfer to the recipient until their immune system is reconstituted, though this transferred immunity is typically short-lived.^(3,6,17,22)^

There is no consensus on the start date and number of doses administered for bone marrow transplant patients due to the lack of studies on the immunogenicity and efficacy of vaccines. In general, several services begin vaccination three to six months after transplantation.^(4,6,23)^

Specially recommended vaccines: Whenever possible, PCV20 or PCV15 should be used and if not possible, PCV13 with or without sequential use of the pneumococcal polysaccharide vaccine (PPV23). When PCV20 is used, there is no indication for a sequential regimen with PPV23. Haemophilus influenzae type b, meningococcal conjugate ACWY, meningococcal B, hepatitis A and B, influenza (preferably the high-dose vaccine [HD] in people aged 60 and over), COVID-19, herpes zoster, HPV, dual bacterial adult-type (Td) or triple bacterial acellular adult-type (Tdap) and RSV from 60 years.^(3,4)^

There are two RSV vaccines available: Arexvy® (GsK) and Abrysvo® (Pfizer). Abrysvo® is licensed by Anvisa for people with certain chronic medical conditions from 18 to 59 years of age. It is also the only one licensed for pregnant women.

Unavailable in CRIEs: PCV15 and PCV20; meningococcal B; herpes zoster; HD influenza, HPV9 and RSV.^(3,4)^

Live attenuated vaccines – yellow fever, triple viral (measles, mumps and rubella), tetraviral (measles, mumps, rubella and varicella) and dengue – may be recommended 12-24 months after transplantation, but in the presence of severe immunosuppression, they are contraindicated. In the case of moderately immunocompromised patients, clinical parameters and epidemiological risk should be assessed for decision making regarding recommendation of these vaccines.^(3,4)^

## How important is it to vaccinate people in contact with the patient and what precautions should we take with vaccination?

Vaccination of people in contact with the patient is important and especially recommended to protect the patient from unnecessary exposure to risk situations, especially when patients have not yet been released for active immunization or even if they have been vaccinated, since there is no certainty regarding the adequacy of the immune response. Some of them are available at CRIEs for this situation and for others related to immunosuppression.^(3,4,6)^

## Final considerations

Vaccinating people who are or will become immunosuppressed, as well as their contacts, offers protection against several infections, increasing the chance of successful treatment of the underlying disease. The increasing implementation of vaccines for several infectious agents is one of the important public health measures, even more relevant for the immunocompromised population. Although the provision of many immunobiologicals by CRIE allows access to the entire population, it is important to emphasize that several vaccines recommended by Febrasgo and other Societies, such as the SBIm, are only available in the private network.
